# Endometrial organoid and stromal cultures demonstrate donor-derived cellular origin of the endometrium after uterus transplantation

**DOI:** 10.3389/fcell.2026.1830798

**Published:** 2026-05-12

**Authors:** Lisa M. Meisl, Ingrid A. Teufel, Anjali Ralhan Singh, Annette Staebler, Melanie Henes, Sara Y. Brucker, Katharina Rall, André Koch

**Affiliations:** 1 Research Institute for Women’s Health, University of Tübingen, Tübingen, Germany; 2 Institute of Pathology, University of Tübingen, Tübingen, Germany; 3 Department of Women’s Health, University of Tübingen, Tübingen, Germany

**Keywords:** bone marrow-derived stem cells, endometrium, genotype, MRKH syndrome, patient-derived organoids, STR profiling, uterus transplantation

## Abstract

The human endometrium undergoes cyclical regeneration during the menstrual cycle, a process thought to be driven primarily by multipotent stem cells located within the basalis layer. Previous studies have proposed that bone marrow–derived cells (BMDCs) may contribute to endometrial regeneration; however, this hypothesis remains controversial due to methodological limitations in distinguishing hematopoietic cells from resident endometrial epithelial or stromal populations. To investigate the potential contribution of BMDCs to endometrial regeneration, we used uterus transplantation (UTx) as a unique donor–recipient model and established patient-derived 3D endometrial organoid (PDO) and 2D stromal cell cultures from UTx recipients. These culture systems enable selective expansion of epithelial and stromal compartments while excluding hematopoietic cells, thereby allowing accurate assessment of cellular origin through donor–recipient genotyping. Samples were collected from four UTx cases spanning up to 7 years, including biopsies obtained before and after pregnancy as well as tissue collected at uterus explantation. Short tandem repeat (STR) profiling was performed on genomic DNA isolated from donor and recipient blood, endometrial organoids, and stromal cell cultures to determine cellular genotype. Endometrial epithelial organoids derived from all UTx cases exhibited consistent morphological features comparable to healthy endometrium and demonstrated long-term expandability. STR analysis revealed complete concordance between the donor genotype and both epithelial and stromal cells of the transplanted uteri, with no evidence of recipient-derived cells or BMDC contribution to the endometrium. These findings indicate that the cellular composition of the endometrium in transplanted uteri remains donor-derived over extended follow-up, including after pregnancy. Together, these results provide strong evidence against a substantial contribution of BMDCs to endometrial regeneration and establish patient-derived endometrial organoid and stromal cultures as powerful model systems for investigating endometrial biology and regenerative mechanisms.

## 1 Introduction

The endometrium, constituting the innermost mucosal layer of the uterus, is composed of mesenchymal stromal cells and endometrial glandular structures. Throughout each menstrual cycle, dynamic changes lead to the breakdown, differentiation and regeneration of the endometrium (Holdsworth-Carson et al., 2023). This cyclic process of tissue regeneration and growth is critical for establishing an optimal environment for embryo implantation and development, thereby playing a pivotal role in mammalian reproduction. This constant remodeling implies that multipotent endometrial stem cells, located in the basalis layer of the endometrium, are involved to facilitate uterine repair (Critchley et al., 2020; Jabbour et al., 2006). Whereas tissue stem cells are present in the basal layer, there is evidence suggesting that bone marrow may serve as an exogenous reservoir of endometrial stem cells (Chan et al., 2004; Lee and Yi, 2018). The first observation for the presence of bone marrow-derived cells (BMDCs) inside the uterine cavity was postulated in 2004 by Taylor, indicating that BMDCs can engraft and integrate into recipient endometrium. Analysis was conducted on endometrial biopsy samples obtained from patients who underwent HLA-mismatched allogeneic bone marrow transplantation. The investigation utilized isolated DNA and formalin-fixed paraffin-embedded (FFPE) tissue samples to detect the presence of the sex-determining region of the Y chromosome (SRY) and the donor HLA type through immunohistochemistry and polymerase chain reaction (PCR) techniques. Thereby, donor-derived BMDCs were identified in the endometrium of HLA-mismatched allogenic bone marrow transplant patients (Taylor, 2004). Subsequent murine studies involving male-to-female transplantation revealed migration and incorporation of donor-derived BMDCs into the female uterus, highlighting their potential role as progenitor cells contributing to endometrial regeneration (Du and Taylor, 2007). Further investigations confirmed the presence of BMDCs within endometrial glands and stromal compartments of bone marrow transplant recipients, although these donor-derived cells were present in limited quantities, raising doubts on their functional contribution as stem cells to endometrial differentiation (Ikoma et al., 2009). However, the interpretation of these findings is complicated by methodological limitations, particularly the inability to distinguish between resident hematopoietic cells and true endometrial epithelial or stromal cell repopulation. This small number of donor-derived cells, combined with technical challenges in definitively excluding hematopoietic contamination, raises questions about whether BMDCs truly contribute to endometrial regeneration or merely represent transient immune cell infiltration (Sasson and Taylor, 2008). One limitation of studying endometrial physiology, particularly with regard to stem cell contributions, has been the lack of *in vitro* models capable of definitively excluding hematopoietic contamination. Three-dimensional (3D) organoid culture models offer a promising solution, providing new insights by replicating the structural and functional characteristics of native tissue (Huch and Koo, 2015). Endometrial organoids have therefore emerged as a valuable *in vitro* model for investigating endometrial physiology, including the cellular and molecular mechanisms that drive regenerative processes and dynamic tissue remodeling (Boretto et al., 2017; Jabri et al., 2025). When cultured within scaffolds that mimic the stem cell niche, endometrial organoids replicate key features of normal uterine epithelial function and display hormonally responsive behavior. Their ability to recapitulate the complex biology of the endometrium makes them a powerful tool for studying the pathogenesis of diseases affecting the female reproductive tract. Furthermore, patient-derived organoids (PDO) provide opportunities for personalized medicine approaches by enabling disease modeling and therapeutic testing in a patient-specific context (Boretto et al., 2017; Kopper et al., 2019; Jabri et al., 2025) A significant condition affecting the uterus is uterine factor infertility (UFI) (Brännström et al., 2014; Grimbizis, 2001), which is estimated to impact 3%–5% of women worldwide. UFI may arise from congenital conditions such as Mayer-Rokitansky-Küster-Hauser (MRKH) syndrome (Herlin et al., 2014). A characteristic feature of MRKH syndrome is the variable presence of uterine rudiments, which may appear unilaterally or bilaterally and exhibit considerable heterogeneity in size and morphology. Although often asymptomatic, some uterine rudiments contain functional endometrial tissue, which can cause cyclical pelvic pain or endometriosis due to menstrual-like bleeding. This frequently necessitates early laparoscopic removal (Brucker et al., 2022; Brännström et al., 2015). Characterized by uterine and upper vaginal agenesis, MRKH syndrome has limited the reproductive options for affected individuals to adoption or gestational surrogacy. However, following decades of preclinical research, the first successful uterus transplantation (UTx) resulting in live birth was achieved in 2014 (Brännström et al., 2015; Brännström et al., 2010). This milestone expanded the field of transplantation surgery and provided women with UFI the possibility of carrying and giving birth to their own child (Brännström et al., 2021). Beyond its transformative impact on UFI treatment, UTx offers unique research opportunities in stem cell biology. The comprehensive donor-recipient matching process involves rigorous pre-screening and extensive post-transplantation monitoring through *in vitro* fertilization, pregnancy, delivery, and eventual graft explantation. Strategic collection of endometrial biopsies at defined timepoints, combined with the establishment of patient-derived organoids, enables systematic investigation of the origins and dynamics of endometrial cell populations within transplanted uteri. Unlike previous studies that primarily reported the presence of BMDCs within the uterine cavity without resolving their tissue-specific contribution, our study investigated the cellular origin of endometrial epithelial and stromal components directly. We employed 3D endometrial organoids derived exclusively from epithelial cells and 2D stromal cell cultures from four UTx patients, both with well-defined cellular compositions that exclude hematopoietic contamination, enabling precise cellular characterization and determination of genetic origin by STR profiling.

## 2 Materials and methods

### 2.1 Patient cohort and sample collection

Endometrial biopsies and tissue samples were obtained from women who had undergone an UTx at the University Hospital of Tübingen after informed written consent. For the biopsies, a Pipelle (1111000, Prodimed, France) was used. All samples were collected in sterile containers containing advanced DMEM/F-12 (advDMEM/F12; 12634010, Thermo Fisher Scientific, United States). We received samples from uterus donors and uterus recipients (Table 1). In total we included four UTx cases with a total of eight blood samples (donor and recipient), twelve endometrial samples (all recipient), five myometrial samples (all recipient), and one uterine rudiment (recipient).

**TABLE 1:** Clinical characteristics of uterus transplantation (UTx) donors and recipients.

Sample ID	Age at UTx	Relation	UFI	Obstetric history
Case I - donor	56	Mother	—	G3 P3 (1x C-section, 2x NSVD)
Case I - recipient	32	Daughter	MRKH type I	G1 P1 (1x C-section)
Case II - donor	32	Sister	—	G2 P2 (2x NSVD)
Case II - recipient	35	Sister	MRKH type I	G1 P1 (1x C-section)
Case III - donor	47	Mother	—	G2 P2 (2x NSVD)
Case III - recipient	23	Daughter	MRKH type I	G1 P1 (1x C-section)
Case IV - donor	46	Mother	—	G4 P4 (4x NSVD)
Case IV - recipient	23	Daughter	MRKH type I	G3 P2 (2x C-section)

Donor–recipient pairs for four UTx cases are shown, including age at transplantation, relationship, underlying cause of uterine factor infertility (UFI), and obstetric history. Gravidity (G) and parity (P) are indicated, with mode of delivery specified (C-section; NSVD, normal spontaneous vaginal delivery). MRKH: Mayer–Rokitansky–Küster–Hauser syndrome.

### 2.2 Processing of endometrial samples

Endometrial biopsy and tissue samples were placed in a sterile culture dish and divided into different parts using a scalpel and processed for a) cryopreservation (fresh frozen in liquid nitrogen), b) formalin fixation for paraffin embedding and c) further processed for culture set-up.

### 2.3 Establishment of endometrial organoids

Endometrial biopsies or tissue samples from UTx recipients were minced into small fragments (0.5–2 mm^3^) using a scalpel and collected in a 50 mL reaction tube containing advDMEM/F12. The suspension was filtered through a 300 μm cell strainer (43-50300-50, Pluri Select Life Science, Germany). The flow-through was immediately centrifuged at 500 g for 10 min, and the resulting cell pellet was resuspended in advDMEM/F12. Tissue pieces retained on the filter were recovered and enzymatically digested with collagenase S (1-mg/mL; 17101015, Thermo Fisher Scientific, United States) in the presence of ROCK inhibitor Y-27632 (10 μM; TMO-T1725, TargetMol, United States) for 45 min at 37 °C on an orbital shaker at 800 rpm. Enzymatic digestion was attenuated by the addition of advDMEM/F12 and the suspension was filtered again through a 300 μm cell strainer. The flow-through was centrifuged at 500 g for 10 min and the resulting cell pellet resuspended in advDMEM/F12. The desired amount of cell suspension was then mixed with BME (Basement Membrane Extract Type II, 3533-001-2, Bio-Techne, United States) at a ratio of 65% BME to 35% cell suspension. 20 μL droplets were plated onto pre-warmed 48-well plates and incubated upside-down at 37 °C with 5% CO_2_ to allow matrix solidification. Afterwards, endometrial culture medium (Supplementary Table S1) was added to each well and renewed every 3 days.

Residual cells and tissue pieces (recovered from cell strainer) were resuspended in Recovery™ Cell Culture Freezing Medium (12648-010, Thermo Fisher Scientific, United States), transferred to cryo-vials and then cooled down in CoolCell™ LX Freezing Containers (CLS432002, Merck, Germany) in a −80 °C freezer. The next day, vials were transferred for long-term storage to liquid nitrogen tanks. The tissue sample was either cryogenically preserved for long-term storage or set up in 2D in a T75 flask with advDMEM/F12 + 10% FCS (fetal calf serum, 10270-106, Thermo Fisher Scientific, United States).

### 2.4 Passaging/cryopreservation of organoid cultures

For passaging or cryopreservation, BME domes with endometrial organoids were resuspended in ice-cold PBS (P04-36500, PAN Biotech GmbH, Germany) containing 10 µM Y-27632, transferred to a 15 mL tube and centrifuged at 500 g for 5 min. The pellet was incubated with 1 mL TrypLE™ (TrypLE Express Enzyme, 12604013, Thermo Fisher Scientific, United States) for 7–10 min at 37 °C and afterwards pipetted up and down 20 times. The suspension was then centrifuged at 500 g for 5 min and the cell pellet either set up in BME domes (as described above) and/or cryogenically preserved in Recovery™ Cell Culture Freezing Medium and stored in liquid nitrogen.

### 2.5 DNA isolation

Genomic DNA from patient blood, 3D endometrial organoid or 2D stromal cell samples, was isolated according to the QIAGEN QIAmp DNA Mini Kit (56304, QIAGEN, Netherlands). Culture duration and passages of samples used for DNA isolation are depicted in Table 2. DNA concentrations were measured with the Nanodrop™ One^C^ spectrometer (Thermo Fisher Scientific, United States).

**TABLE 2:** Overview of sample collection and processing in UTx cases.

UTx ID	Sample ID	TF	Days after UTx	Type	Origin	Culture	Time of DNA sampling
Case I	UTx #02	T1	426	Biopsy	Endo	3D-PDO (Endo)	P6 (62 days)
	UTx #06	T2	832	Biopsy	Endo	3D-PDO (Endo)	P6 (64 days)
	UTx #14	T3	1,511	Biopsy	Endo	3D-PDO (Endo)	P2 (16 days)
	UTx #16	T3	1,511	Tissue	Myo	2D culture (Myo)	P2 (13 days)
	UTx #17	T3	1,511	Tissue	Endo	3D-PDO (Ut. Rud.)	P3 (26 days)
Case II	UTx #03	T1	142	Biopsy	Endo	3D-PDO (Endo)	P9 (76 days)
	UTx #09	T3	708	Biopsy	Endo	3D-PDO (Endo)	P5 (51 days)
	UTx #10	T3	708	Tissue	Endo	3D-PDO (Endo)	P6 (56 days)
	UTx #11	T3	708	Tissue	Endo	3D-PDO (Endo)	P5 (49 days)
	UTx #12	T3	708	Tissue	Myo	2D culture (Myo)	P2 (23 days)
	UTx #13	T3	708	Tissue	Myo	2D culture (Myo)	P2 (23 days)
Case III	UTx #04	T2	1,015	Biopsy	Endo	3D-PDO (Endo)	P8 (84 days)
Case IV	UTx #05	T2	1,279	Biopsy	Endo	3D-PDO (Endo)	P4 (51 days)
	UTx #08	T2	1,719	Biopsy	Endo	3D-PDO (Endo)	P4 (51 days)
	UTx #21	T3	2,595	Tissue	Endo	3D-PDO (Endo)	P5 (37 days)
	UTx #22	T3	2,595	Tissue	Endo	3D-PDO (Endo)	P3 (30 days)
	UTx #24	T3	2,595	Tissue	Myo	2D culture (Myo)	P2 (25 days)
	UTx #25	T3	2,595	Tissue	Myo	2D culture (Myo)	P2 (25 days)

Listed are Case ID, sample ID, time frame, days post-transplantation, sample type, tissue origin, culture model, and passage number with total number of days at DNA sampling; TF, Time frame; Passage; Endo, Endometrium; Myo, Myometrium; Ut. Rud., Uterine rudiment.

### 2.6 STR profiling

For the authentication of different genotypes, STR profiling was performed by Multiplexion GmbH, Heidelberg using the standardized guideline ANSI/ATCC ASN0002-2011 for cell line authentication. Therefore, > 15 ng/μL of isolated DNA in a total volume of 40 µL were sent to Multiplexion GmbH, Heidelberg. For cell line authentication of each DNA sample, a PCR reaction with specific primers targeting different STRs, followed by a capillary electrophoresis was performed. Sixteen different STR markers were used to compare short tandem repeats at specific loci on different chromosomes: CSF1PO, D2S1338, D3S1358, D5S818, D7S820, D8S1179, D13S317, D16S539, D18S51, D19S433, D21S11, FGA, TH01, TPOX, vWA, und Amelogenin.

### 2.7 Ethic statement

The study was conducted according to the guidelines of the Declaration of Helsinki and was approved by the Ethics Committee of the University of Tübingen (Ethics approval 150/2018BO2) and is compliant with all relevant ethical requirements for research involving human participants. The Tübingen living-donor UTx clinical program received initial approval from the University of Tübingen and the Ethics Committee of the University of Tübingen (project identification code 211/2016A). All tissue biopsies were obtained from patients after informed written consent.

## 3 Results

The endometrium, which constitutes the innermost lining of the uterus, undergoes cyclical regeneration, a process primarily driven by multipotent stem cells localized within its basalis layer (Jabbour et al., 2006; Critchley et al., 2020). Recent studies have suggested that bone marrow may serve as an extrinsic reservoir of endometrial stem cells (Taylor, 2004; Du and Taylor, 2007; Ikoma et al., 2009). To further investigate the potential contribution of BMDCs to endometrial regeneration, we utilized well-defined patient-derived endometrial organoids and stromal cultures obtained from four UTx patients. This approach enables the isolated cultivation of stromal and epithelial cells from the uterine cavity while excluding hematopoietic cells, which would otherwise be of recipient origin. These cell culture models therefore provide a robust platform for investigating the cellular origin of endometrial tissue and the dynamics of potential stem cell migration.

**FIGURE 1:**
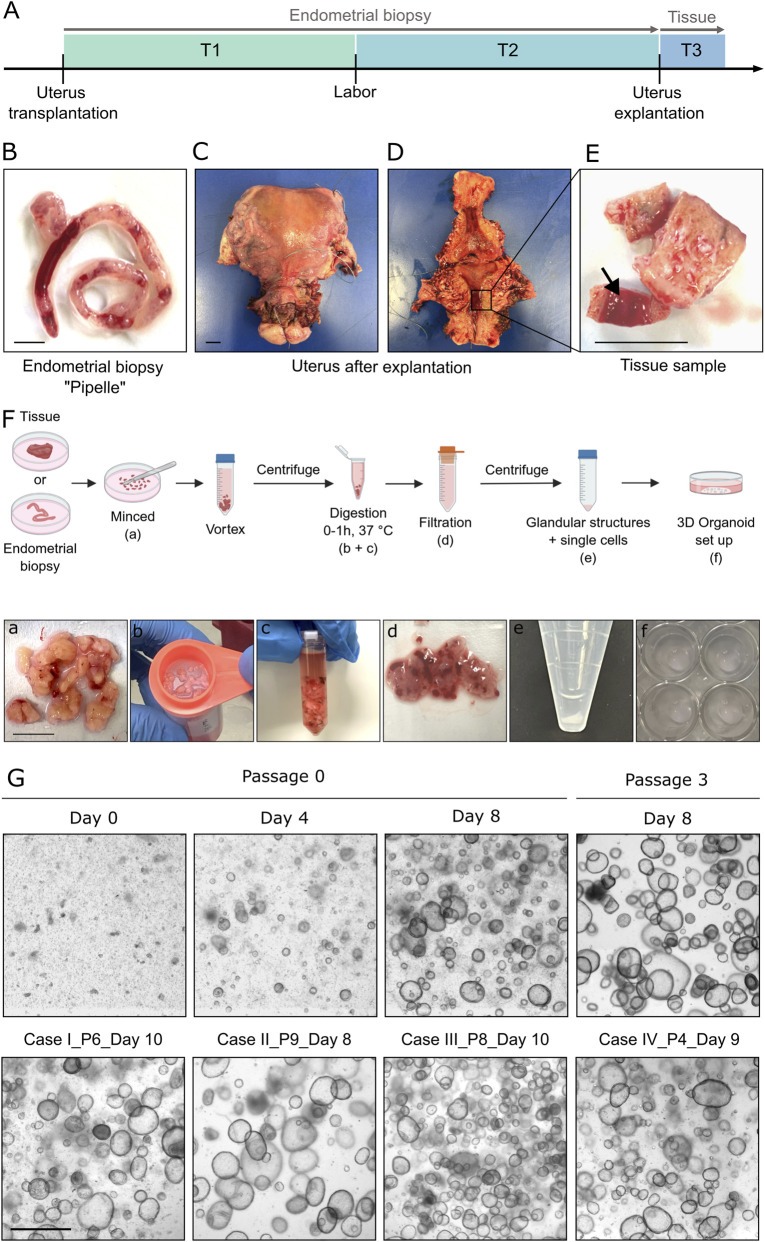
Collection of endometrial biopsies and uterine tissue samples at different time points. **(A)** Timeline demonstrating the possible time points at which endometrial biopsy and tissue samples can be obtained. Collection of endometrial biopsy samples is possible from the recipient prior to *in vitro* fertilization (T1), after birth (T2), or directly before uterus explantation. Additionally, uterine tissue samples (endometrium + myometrium) can be collected after uterus explantation (T3). **(B)** Endometrial biopsy sample obtained by a Pipelle. **(C)** Explanted uterus of a uterus transplanted woman before segmentation. **(D)** Explanted uterus after coronal section, unfolded. **(E)** Excised section of myometrium and endometrium from the uterus (approximately 1–2 cm^3^); the endometrium is indicated by the arrow. Scale bar: 1 cm. **(F)** Scheme illustrating the procedure for establishing endometrial organoids derived from endometrial biopsy or tissue samples. Minced pieces (a) can be digested with collagenase S (b + c) and filtered (d). The flow-through of each section can be set up in 3D (e + f). Created with Biorender.com. **(G)** Brightfield images of exemplary endometrial organoids established from endometrial biopsy samples at passage 0, cultured for 8 days. Single cells and glandular structures were used to establish the organoids. The right part shows the same culture at day 8 of passage 3. Second row demonstrating bright-field images of endometrial organoids from all four UTx cases. T1-T3, Timepoints of sample collection; P, Passage; Scale bar **(A–F)** 1 cm; Scale bar **(G)** 1,000 µm.

For this purpose, biopsy and tissue samples from four UTx recipients were processed (patient characteristics in Table 1). An overview of sample origin and collection time points is provided in Table 2. The interval between transplantation and sample collection ranged from 142 to 2,595 days, encompassing multiple endometrial regeneration cycles and five pregnancies across the cohort of four UTx cases. Endometrial biopsies were collected either prior to *in vitro* fertilization, designated as time frame T1 (Figure 1A, T1 (green)) or after childbirth, referred to as time frame T2 (Figure 1A, T2 (light blue)). In addition, endometrial biopsies (Figure 1B) and tissue samples (Figures 1C–E) were obtained at the time of uterus explantation, classified as time frame T3 (Figure 1A, T3 (dark blue)). For organoid establishment, minced tissue was enzymatically digested, filtered, and embedded for 3D culture (Figure 1F, steps a–f). Long-term expandable patient-derived organoid models were established with a 100% success rate from all samples, independent of sample type or collection time within the UTx process (Figure 1G). We established and analyzed 3D endometrial organoids from all four cases and 2D stromal cell cultures from three. Sampling distribution varied across cases: Case I (T1, T2, T3), Case II (T1, T3), and Case III (T2 only). Myometrial samples were collected at T3 from Cases I, II, and IV (Table 2). Case III had not undergone explantation, precluding stromal culture generation. Endometrial organoids from all UTx patients appear to exhibit uniform morphological characteristics irrespective of sampling timepoint, forming cystic structures with columnar epithelial lining and retaining proliferative potential through extended passaging. (Figure 1G; Supplementary Figure S1).

**FIGURE 2:**
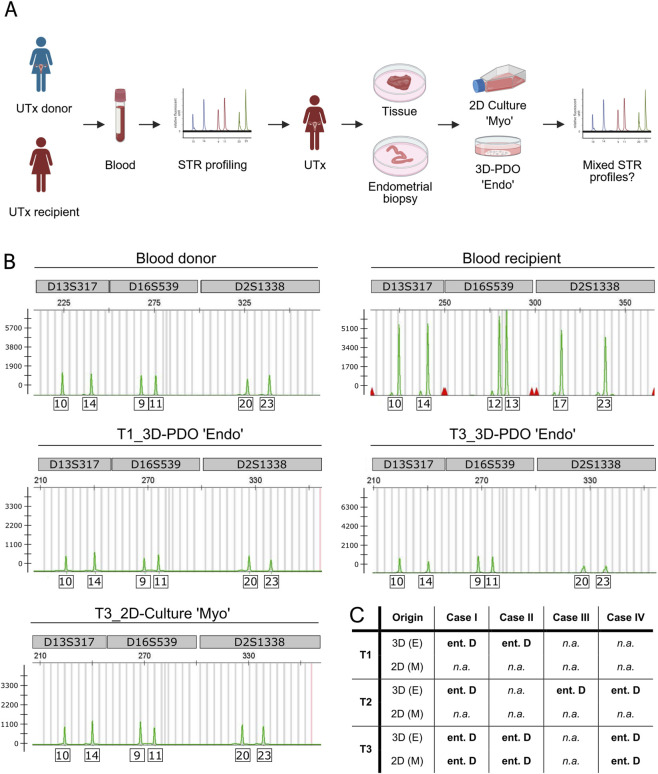
STR profiles of endometrial organoid and 2D stromal cell cultures derived from endometrial biopsy and tissue samples correspond entirely to the STR profiling genotype of the UTx donor. **(A)** Graphical representation of the procedure for STR profiling of blood, 3D organoids and 2D stromal cells. Created with Biorender.com. **(B)** Exemplary STR profiles with allele determination by PCR amplicons of different human STR loci for genomic DNA. Genomic DNA was either extracted from blood of UTx donor and recipient or from patient-derived endometrial organoids (PDO) derived from endometrial biopsy (T1) or from endometrial tissue sample (T3). Additionally, genomic DNA was isolated from 2D stromal cell cultures derived from myometrium (T3). Y-axis represents the relative fluorescent units (RFU); the x-axis represents the base pair values. The exemplary STR loci are localized on different chromosomes: D13S317 - chromosome 13; D16S539 - chromosome 16; D2S1338 - chromosome 2. **(C)** Table representing 3D endometrial organoid and 2D stromal cell culture samples for three different timepoints of four UTx cases. According to STR profiling analysis, isolated DNA originated entirely to the UTx donor genotype. Four different UTx cases; “Myo”/M, Myometrium; “Endo”/E, Endometrium; PDO, Patient-derived organoids; T1–T3, Timepoints of sample collection; D, Donor; ent., Entirely; n. a., Not available.

To determine the genetic origin of endometrial samples, genomic DNA was isolated from recipient and donor blood samples, as well as from patient-derived endometrial organoids (PDO) and myometrial mesenchymal stromal cells cultured from endometrial biopsies and uterine tissue samples. The culture duration and sample passages used for DNA isolation are provided in Table 2. STR profiling was selected as the analytical method for genomic characterization. Multiplexed PCR amplification followed by capillary electrophoresis enabled allele determination at multiple human STR loci (Figure 2A). Representative STR profiles are presented in Figure 2B with allele determination by PCR amplicons across three exemplary human STR loci, located on different chromosomes: D13S317 (chromosome 13); D16S539 (chromosome 16); D2S1338 (chromosome 2) (Figure 2B). The number of STRs of each allele of the STR loci, which are highly polymorph, were used to determine the genotype for each sample. Notably, the DNA isolated from 3D endometrial organoid or 2D stromal cell samples, collected at various time points, entirely matches with the number of STRs present in the donor blood (Figure 2C). This STR profiling approach was applied across all four UTx cases, encompassing eight blood samples (four donors and four recipients), twelve endometrial organoid samples, five mesenchymal stromal cell culture samples and one uterine rudiment organoid sample (Table 2; Supplementary Table S2). Across all four UTx cases and time points, STR profiles corresponded exclusively to donor DNA, confirming that the cellular composition of epithelial cells and stromal cells (excluding other cell populations) in all UTx cases comprises cells derived exclusively from the donor uterus, with no contribution from the recipient. Importantly, across all UTx cases and time points, no evidence for recipient-derived endometrial stem cell migration or differentiation into epithelial endometrial cells within the transplanted uterus was found (Supplementary Table S2).

To further validate that STR profiling could distinguish between heterogeneous cellular populations (endometrial epithelial cells, myometrial cells, hematopoietic cells, and immune cells), single-cell suspensions from endometrial tissue digests were also analyzed for three out of four UTx cases. The STR profiling analyses revealed a mixture of donor and recipient DNA, demonstrating that such heterogeneous samples, especially containing hematopoietic cells, are unsuitable for precise determination of endometrial cell origin (Supplementary Table S3). Using defined cellular populations, in this case, 3D organoids comprising exclusively epithelial cells, as well as 2D stromal cells, emphasizes the importance of separating cellular compartments and excluding any hematopoietic cells present in the originating tissue. Therefore, patient-derived endometrial organoids, and 2D stromal cells represent a robust model for assessing potential BMDC migration into the endometrium.

**FIGURE 3:**
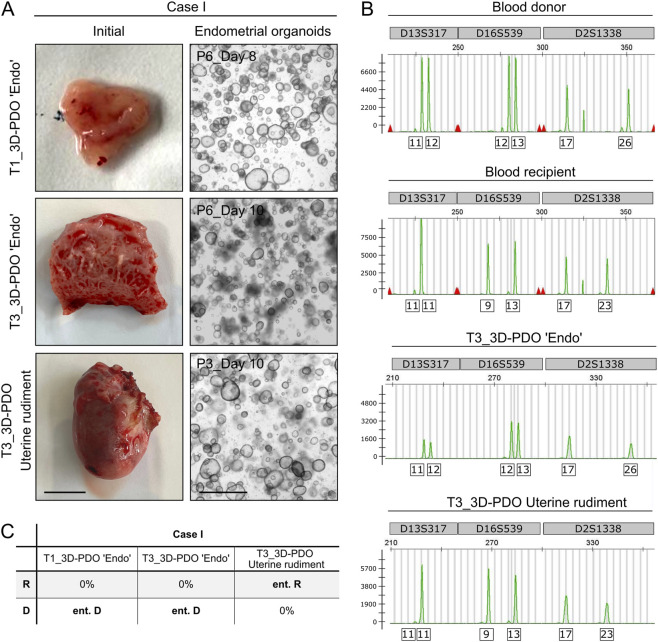
Coexistence of two genetically distinct endometria in uterine rudiment and uterus emphasizes no evidence for stem cell migration during the UTx process. **(A)** Bright-field images of endometrial organoids derived from endometrial biopsy (T1), endometrial tissue (T3) or uterine rudiment samples, exemplary for Case I. Endometrial organoids appear morphologically similar among the different samples. Scale bar tissue: 1 cm; scale bar organoids: 1,000 µm. **(B)** STR profiles for Case I recipient blood and donor blood, as well as endometrium from T3 and the uterine rudiment. Y-axis represents the relative fluorescent units (RFU); the x-axis represents the base pair values. The exemplary STR loci are localized on different chromosomes: D13S317 - chromosome 13; D16S539 - chromosome 16; D2S1338 - chromosome 2. **(C)** Table showing STR data analyses for Case I, identifying the uterine rudiment originating entirely to the UTx recipient genotype, in comparison to the 3D endometrial samples originating entirely to the UTx donor genotype. T1 + T3, Timepoints of sample collection; D, Donor; R, Recipient; ent., Entirely. Scale bar (initial): 1 cm; Scale bar (organoids): 1,000 µm.

These findings were further supported by a unique clinical scenario in Case I. Patients with MRKH syndrome undergoing UTx typically lack uterine rudiments, often due to prior laparoscopic excision. However, Case I represented an exception in which a residual uterine rudiment was still present at the time of uterus explantation and was removed concomitantly with the transplanted uterus. We have previously demonstrated that endometrial organoids can be successfully established from uterine rudiment-derived endometrial tissue, exhibiting morphological features comparable to those derived from endometrial biopsies and tissue samples (Figure 3A; (Brucker et al., 2022)). STR profiling confirmed that DNA isolated from the uterine rudiment-derived culture models matched the recipient genotype (Supplementary Table S2), whereas DNA obtained from endometrial biopsy- and uterine tissue sample-derived models matched the donor genotype (Figures 3B,C). This case uniquely demonstrated the coexistence of two genetically distinct endometria during the UTx process: one within the donor uterus and the other within the recipient’s residual uterine rudiment. These findings further support the absence of recipient stem cell migration into the donor endometrium. Collectively, our results provide no evidence that BMDCs from UTx recipients migrate to, or contribute to, the cellular composition of the endometrium in transplanted uteri across the studied cases.

## 4 Discussion

The human endometrium undergoes continuous structural and functional changes throughout the menstrual cycle in response to hormonal regulation. These processes include menstruation, proliferation, and secretory differentiation, all of which are tightly regulated. The primary purpose of these cyclical changes is to establish a microenvironment suitable for embryo implantation following decidualization (Holdsworth-Carson et al., 2023; Wong et al., 2025). This remarkable regenerative capacity underscores the critical role of stem cells in maintaining uterine function. In this study, we investigated the potential contribution of BMDCs to endometrial regeneration within the stem cell niche, using UTx as a unique experimental model.

Multipotent endometrial stem cells located in the basalis layer are believed to drive reconstruction of the *functionalis* layer during each menstrual cycle (Padykula, 1991; Leyendecker, 2002; Santamaria et al., 2018). The hypothesis that BMDCs contribute to endometrial regeneration originated from studies identifying donor-derived cells in endometrial biopsies from HLA-mismatched allogeneic bone marrow transplant recipients (Taylor, 2004). Subsequent studies proposed that BMDCs may differentiate into functional endometrial components (Ikoma et al., 2009) and contribute to endometrial vascularization (Mints et al., 2007). However, the cellular identity of endometrium-contributing BMDCs remains unclear, particularly whether these cells belong to hematopoietic or mesenchymal stem cell populations.

Previous studies assessing BMDC contributions used isolated genomic DNA or FFPE tissue samples to detect donor-derived genetic markers such as the sex-determining region of the Y chromosome (SRY) gene or donor HLA types using polymerase chain reaction (PCR) and immunohistochemical (IHC) approaches. However, the use of IHC for identifying BMDCs in endometrium tissue presents important limitations, particularly due to potential cellular overlap within tissue sections. Processing heterogeneous tissue samples containing epithelial, stromal, immune, and hematopoietic cells carries a substantial risk of cross-contamination between distinct cellular populations (Wong et al., 2025). To exclude this risk, we established endometrial organoid models that recapitulate the complex physiological dynamics of the epithelial cell compartment *in vitro* (Snider et al., 2011). Serial passaging of organoid cultures promotes the gradual loss of contaminating cell populations, including hematopoietic cells, thereby enabling the exclusive cultivation of epithelial cells. Organoids are *in vitro* models capable of recapitulating key functional properties of organs and tissues (Clevers, 2016). These systems exhibit self-organizing capacity, long-term expandability for biobanking, and phenotypic characteristics that closely resemble their tissue of origin (Turco et al., 2017). As such, the organoid technology has emerged as a valuable platform for disease modeling and applications in precision medicine (Ivell, 2017; Huch and Koo, 2015).

UTx represents a clinical field that can particularly benefit from advances in endometrial organoid research. This procedure offers a potential treatment option for women affected by permanent UFI (Brännström et al., 2014; 2010). By combining the advantages of endometrial organoids–which consist exclusively of epithelial cells and exclude hematopoietic and immune cells–with samples obtained from UTx, we established a novel model system to investigate endometrial development, remodeling, and potential stem cell differentiation processes.

To assess the potential migration of BMDCs and their contribution to endometrial regeneration, we established endometrial organoid cultures from patients undergoing or having undergone UTx. Endometrial biopsies obtained using Pipelle sampling were used as the primary source for organoid establishment. This minimally invasive method allows samples to be collected at multiple time points while the transplanted uterus remains *in situ*, with pregnancy representing the only contraindication (Luerti et al., 2019). In our study, organoids were successfully established in all cases, yielding a 100% success rate consistent with our previous work on organoid generation from women with MRKH syndrome (Brucker et al., 2022). Despite its advantages for longitudinal sampling, Pipelle biopsy collection typically retrieves only superficial endometrial tissue, whereas surgical tissue collection allows analysis of the full endometrial composition–including the *stratum functionalis* and *stratum basalis*–as well as the myometrium (Snider et al., 2011). A further limitation is the relatively small sample size of four UTx cases. However, it is important to emphasize that UTx remains an experimental procedure that is still being developed within ongoing clinical trials. With an estimated number of 67 live donor transplantations performed worldwide (Brännström et al., 2026), long-term follow-up studies spanning several years with repeated sampling before and after pregnancy remain restricted to the limited number of cases available at specialized centers, including the Women’s Hospital in Tübingen. To evaluate the potential migration of BMDCs into the endometrium and their possible differentiation into glandular epithelial or mesenchymal stromal cells, we selected STR profiling as our analytical method. STR loci consist of short, highly polymorphic DNA sequences composed of repeating units typically ranging from one to five base pairs (Almeida and Korch, 2023). These sequences are distributed across multiple chromosomal locations throughout the genome and exhibit substantial variability between individuals, enabling precise genetic identification. For this reason, STR profiling is widely used in human identification laboratories due to its high specificity and reliability (Almeida and Korch, 2023; Almeida et al., 2016). Endometrial organoids maintain genetic stability and exhibit complete concordance with their parental endometrial tissue, as confirmed by STR profiling (Zhang et al., 2025). Consequently, those organoids represent a robust *in vitro* model of endometrial epithelial cells that accurately recapitulates the physiological, morphological, and genetic characteristics of native tissue (Zhang et al., 2025).

Importantly, organoid culture conditions selectively support the expansion of epithelial stem and progenitor cells, while non-epithelial cell populations are gradually lost during serial passaging. This feature enables genetic analysis of a purified epithelial cell population and minimizes the risk of contamination by hematopoietic or immune cells. Although extremely rare events involving recipient-derived cells cannot be entirely excluded and may fall below the detection threshold of STR profiling, such occurrences are likely negligible. Moreover, if recipient-derived epithelial cells were present within the transplanted endometrium, they would be expected to expand during organoid establishment and passaging and therefore become detectable through STR analysis. The absence of recipient-derived genotypes in all analyzed organoid cultures therefore further supports the conclusion that such cells are either absent or present only at extremely low frequencies. While approaches such as single-cell genotyping (Marečková et al., 2024) or fluorescence-activated cell sorting (FACS)–based lineage analyses (Schwalie et al., 2024) could theoretically provide additional resolution for detecting rare cell populations, the organoid-based strategy used in this study already enables selective expansion and genetic characterization of epithelial cells and is therefore well suited to address the question of epithelial cell origin.

Using STR profiling, we traced the genetic origin of cells within patient-derived endometrial organoids by comparing donor and recipient genetic markers. All analyzed endometrial epithelial cells matched the donor genotype rather than the recipient genotype, indicating that BMDCs show no detectable contribution to the epithelial stem cell population of transplanted uteri. These findings are consistent with previous observations by Cervelló et al., who demonstrated that BMDCs do not incorporate into the recipient endometrial side population (Cervelló et al., 2012). Importantly, the observational period in this study extended up to 7 years and included at least one pregnancy and cesarean section per case, with repeated endometrial biopsies collected during routine graft monitoring, providing multiple opportunities to detect potential recipient-derived cells. Throughout this extended follow-up period, no endometrial epithelial cells of recipient origin were detected. Although BMDCs do not appear to contribute substantially to physiological endometrial regeneration in this context, their potential roles in pathological conditions or regenerative responses remain to be clarified. Overall, our findings provide strong evidence that the epithelial cellular composition of the endometrium in transplanted uteri remains exclusively donor-derived, challenging the hypothesis that BMDCs significantly contribute to physiological endometrial regeneration.

## Data Availability

The original contributions presented in the study are included in the article/Supplementary Material, further inquiries can be directed to the corresponding author.
