# Supplementary material for: Endometrial organoid and stromal cultures demonstrate donor-derived cellular origin of the endometrium after uterus transplantation
Source: Front Cell Dev Biol. 2026 May 12;14:1830798. doi: 10.3389/fcell.2026.1830798 (PMC13201452; doi:10.3389/fcell.2026.1830798)
Supplement: Supplementary file 4 [file Image1.pdf]

Supplementary Material:

Supplementary Figure S1:

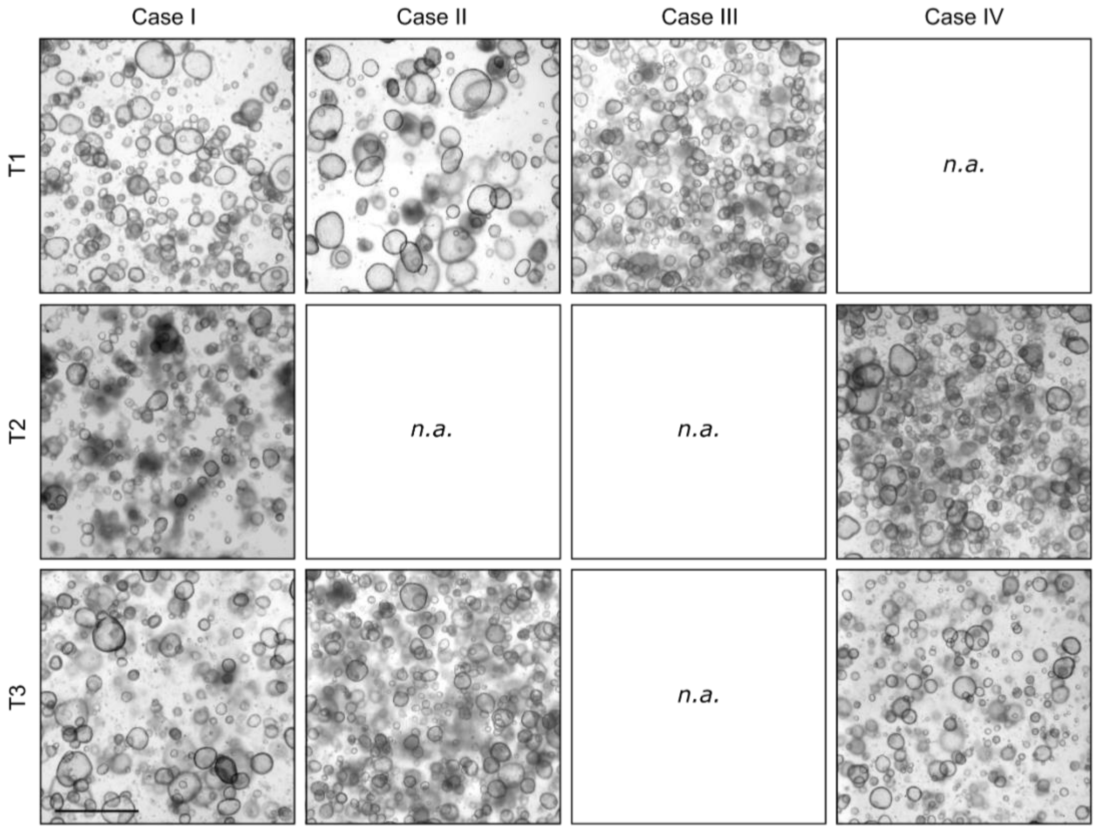

Supplementary Figure S1: Representative images of endometrial organoids across UTx cases and time points. Bright-field images of endometrial organoids from four UTx cases established from endometrial biopsy samples or tissue samples. Organoids, independent of UTx patient and time point of sample collection, appear morphologically similar. T1-T3 = timepoints of sample collection; *n.a.* = not available; Scale bar: 1000 µm.
